# Supplementary material for: Empirical methods for controlling false positives and estimating confidence in ChIP-Seq peaks
Source: BMC Bioinformatics. 2008 Dec 5;9:523. doi: 10.1186/1471-2105-9-523 (PMC2628906; doi:10.1186/1471-2105-9-523)
Supplement: Additional File 1 — A variety of html documents from the USeq web site detailing the available applications, their best usage, output file type descriptions, command line menus, etc. [file 1471-2105-9-523-S1.zip › USeqUserGuides/JavaDoc.zip!/JavaDoc/util/bio/annotation/package-summary.html]

util.bio.annotation


|  |  |  |
| --- | --- | --- |
| **Overview** \| **Package** \| Class \| **Use** \| **Tree** \| **Deprecated** \| **Index** \| **Help** | |  |
| **PREV PACKAGE**   **NEXT PACKAGE** | **FRAMES**    **NO FRAMES**     **All Classes** |  |


---

## Package util.bio.annotation

For representing gene annotation.

**See:**
  
          **Description**

| **Class Summary** | |
| --- | --- |
| **Coordinate** | Base class for holding info about a genomic coordinate. |
| **ExonIntron** | ExonIntron info. |
| **ExportExons** |  |
| **ExportIntergenicRegions** |  |
| **ExportIntronicRegions** |  |
| **GeneGroup** | A GeneGroup is the top organizing level of a "Gene". |
| **GeneRep** | A GeneRep resentation contains a genomic sequence designating a gene broken into various segments. |
| **GenericFeature** | Class to hold user defined gff items like CRMs, enhancers, unpublished single track notation. |
| **ScoredCoordinate** |  |
| **StartStop** | Simple start end object for sorting. |
| **Transcript** | Transcript relevant information, mRNA. |
| **TransGroup** | A TransGroup describes a product derived from a GeneGroup contains one transcript, possibly a translation, and a possibly a set of exons. |
| **Translation** | Translation specific information, protein but referenced with genomic coordinates thus some bits code for protein, some don't physically exist. |

## Package util.bio.annotation Description

For representing gene annotation.

---


|  |  |  |
| --- | --- | --- |
| **Overview** \| **Package** \| Class \| **Use** \| **Tree** \| **Deprecated** \| **Index** \| **Help** | |  |
| **PREV PACKAGE**   **NEXT PACKAGE** | **FRAMES**    **NO FRAMES**     **All Classes** |  |


---
